# Diagnostic Sign of Cancer of the Neck of the Womb; Hepatic Abscess Cured by Injections of Iodized Iodide of Potassium; Medical Responsibility; Erectile Tumor in the Duodenum

**Published:** 1872-12-15

**Authors:** 


Diagnostic sign of Cancer of the Neck of the Womb.—Besides the difference as to consistence between cancer and simple induration of the neck of the uterus, Prof. Spiegelberg, of Breslau, gives the following sign as distinctive: "The mucus in cancer is firmly adherent to the subjacent tumor, and immovable, while the contrary is the case in hyperplastic thickening and induration. In the latter condition, the mucus, under the influence of compressed sponge, becomes looser, softer and thicker. In cancer it invariably remains hard and rigid, and cannot be torn." —*La France Medicale.*

Hepatic Abscess cured by Injections of Iodized Iodide of Potassium.—A merchant, fifty-two years of age, was seized with deep lacerating pain in the right hypochondrium, followed, fifteen days afterward, with swelling in the iliac region of the same side. After two months of intense suffering the tumor opened spontaneously, discharging a large quantity of fetid pus. For three years this patient underwent different modes of treatment without obtaining any amelioration of the local difficulty. His constitution was greatly impaired when Dr. Goldsberry was called in. Dr. G. commenced by injecting into the fistulous sinuses tepid water twice a day; then a solution of four grains of iodine and eight of iodide of potassium in a pint of water. The strength of this solution was gradually increased as the patient was able to bear it. After making use of this treatment for one year, all flow of pus and bile had ceased, the fistulous tracts were entirely closed, and the general condition was very satisfactory. There was no relapse. —*La France Medicale.*

Medical Responsibility.—A woman, arrived at term, dies of cholera; the doctor is arraigned for not having performed the post mortem Cæsarian section. The College of physicians of the Faculty of Vienna, examined as experts, testified that the physician did not arrive till one hour after the decease of the woman, and that the fœtus could not survive the mother longer than five or six minutes. Besides, the annals of science próve that, in cases of cholera, the death of the fœtus always precedes that of the mother. The doctor was discharged from all prosecution.—*La France Medicale.*

Erectile Tumor in the Duodenum.—At a recent session of the Academy of Medicine, at Paris, Dr. Laboulbene reported the following case:

X——, aged about seventy-four years, presenting no symptoms of diathetical diseases, and no infirmities, had passed black stools without manifesting symptoms of a disease of the stomach. He had also vomited blood of a blackish color, and partly coagulated. Attention was directed to the possibility of a stomachic lesion, either cancer or simple ulcer; but, after having interrogated the patient on all the points which might clear up the diagnosis; after having passed in review the commemorative signs, heredity, etc., no certain conclusion could be arrived at. There existed no appreciable tumor in the abdomen, nor any evidence of disease of the liver; the spleen appeared normal; the finger introduced into the rectum revealed nothing unusual. The thoracic organs, lungs, and heart, performed their functions well; the urine contained neither albumen nor sugar. The general symptoms were not those of a disease of the stomach with troubled digestion, obstinate gastralgia, etc. The diagnosis was *ulcerative lesion of the duodenum*, accounting for the several hæmorrhages from the bowels, and the more rare ejection of blood from the stomach.

The patient died a few hours after having presented signs of internal hæmorrhage. The autopsy, conducted with great care, established the perfect integrity, considering the age of the patient, of almost every thoracic and abdominal organ. At a single point the duodenum was the subject of lesion. Below the entrance of the ductus communis choledochus a small oblong tumor of the size of an almond was found placed lengthwise with regard to the intestine. The prominence formed by the morbid growth was plainly visible on the intestine, washed and freed from the blood which had filled it. Examined under water, the mucous membrane covering the tumefaction showed a small ulcerated opening with fringed borders of a reddish brown color. Dr. Laboulbene recognized this opening as the source of the last hæmorrhage. Two other blackish points appeared to be the sites of old orifices of erosions, now healed, through which other hæmorrhages had taken place.

[The minute anatomy of the tumor, clearly establishing its character, is here omitted.]

The conclusions of Dr. L., in the absence of analogous examples, were limited to the following:

1. Erectile tumors exist in the intestinal tube, as at the surface of the external integument.

2. These tumors are developed in the mucous membrane of the intestine.

3. They may give rise to fatal hæmorrhage. —*La France Medicale.*

